# Real-World Economic Outcomes of Patients with Localized Prostate Cancer Treated with External Beam Radiation Therapy or Radical Prostatectomy

**DOI:** 10.36469/001c.163569

**Published:** 2026-07-29

**Authors:** Lawrence Karsh, Shawn Du, Jinghua He, Neal Shore, Kruti Joshi

**Affiliations:** 1 AdventHealth Urology, Denver, Colorado, USA; 2 Johnson & Johnson, Horsham, Pennsylvania, USA; 3 Carolina Urologic Research Center, Myrtle Beach, South Carolina, USA

**Keywords:** localized prostate cancer, radical prostatectomy, external beam radiation therapy, economic outcomes, real-world evidence

## Abstract

**Background:**

External beam radiation therapy (EBRT) and radical prostatectomy (RP) are definitive options for patients with localized or locally advanced prostate cancer (LPC). Limited evidence exists on the post-treatment economic burden in these patients.

**Objective:**

This real-world analysis investigated healthcare costs among Medicare beneficiaries with LPC who received EBRT or RP as initial definitive treatment.

**Methods:**

This is a retrospective, longitudinal cohort study using Surveillance, Epidemiology, and End Results (SEER)–Medicare database. Included were Medicare Fee-For-Service beneficiaries newly diagnosed with LPC at the age of ≥65 years during 2012-2019 and received either EBRT or RP as an initial definitive therapy. After the initial therapy, all-cause and prostate cancer–related costs were summarized and stratified by the earliest observed definitive therapy (RP vs EBRT) and the National Comprehensive Cancer Network risk classification (low/intermediate-risk [LIR-LPC] vs high-risk [HR-LPC]).

**Results:**

Of the 17 066 LPC patients treated with EBRT, 60% (N =10 321) had LIR-LPC and 40% (N = 6745) had HR-LPC. During the follow-up, the mean per-person-per-year all-cause cost was 9837higherintheHR−LPCcohortthantheLIR−LPCcohort(34 441 vs $24 604; *P* < .0001). Of the 9479 patients treated with RP, 43% (N = 4120) had LIR-LPC and 57% (N = 5359) had HR-LPC. The mean per-person-per-year all-cause cost during follow-up was 6829higherintheHR−LPCcohortthantheLIR−LPCcohort(23 820 vs $16 991; *P* < .0001). Across all cohorts, the largest all-cause healthcare cost category was outpatient, followed by inpatient and prescription drug.

**Conclusions:**

This real-world study on healthcare costs in patients with LPC treated with EBRT or RP showed HR-LPC is associated with significant incremental economic burden relative to LIR-LPC. The findings highlight the current unmet need for more cost-effective treatment strategies for HR-LPC.

## BACKGROUND

Excluding non-melanoma skin cancer, prostate cancer is the most common cancer in men in the United States (US), with an estimated 299 010 new cases and 35 250 deaths in 2024.^1^ Although the prostate cancer death rate dropped by approximately half from 1993 to 2013 and has since plateaued,^2^ the overall incidence rate has been increasing by 3% per year, with a 5% annual rise in advanced-stage prostate cancer since 2014.^1^ Consequently, treatment costs for prostate cancer have been increasing rapidly in recent years and ranked as the second-highest out-of-pocket cost nationally in 2019, estimated at $2.26 billion.^3^ This rising economic burden places significant financial strain on both patients and the healthcare system. The burden is expected to increase further as more patients are diagnosed at advanced stages requiring intensive treatment and long-term care.^4^

In the US, localized and locally advanced prostate cancer (LPC), defined as a prostate cancer without distant metastasis, accounted for approximately 70% of prostate cancer cases at diagnosis.^5^ According to the National Comprehensive Cancer Network guideline, an LPC patient is considered high-risk if the Gleason score is at least 8, prostate-specific antigen (PSA) is at least 20 ng/mL, or the T stage is T3a and above.^6^ These high-risk LPC (HR-LPC) patients accounted for approximately 26% of newly diagnosed prostate cancer cases and are at increased risk for recurrence and disease progression following treatment.^7^ Due to their elevated risk, patients with HR-LPC often require more intensive treatments and follow-up, which may contribute disproportionately to overall healthcare utilization and costs.^8,9^

The most widely used initial definitive treatment options for LPC patients are external beam radiation therapy (EBRT) and radical prostatectomy (RP), with the median time from diagnosis to treatment initiation typically ranging from approximately 2 to 3 months.^10–14^ However, limited real-world evidence exists on the long-term economic burden following initial definitive treatment for LPC.

The objective of this study is to examine the healthcare costs among Medicare beneficiaries who were newly diagnosed with LPC and received EBRT or RP as their initial definitive therapy.

## METHODS

### Data Source

The study utilized data from the Surveillance, Epidemiology, and End Results (SEER)–Medicare database. The SEER program includes newly diagnosed cancer cases in up to 21 registry areas, covering approximately 30% of the US population.^15^ SEER collects detailed patient-level data, including demographics, cancer characteristics, survival outcomes, and socioeconomic status at the census tract level.^16^ Medicare is the largest healthcare payer in the US, providing insurance coverage for most individuals aged 65 years and older.^17^ Medicare claims offer longitudinal data on healthcare encounters, treatments, and associated costs.^18^ By linking cancer registries to Medicare claims, the SEER–Medicare database provides a comprehensive, population-level dataset that enables the analysis of real-world treatments, outcomes, and healthcare resource utilization and cost among cancer patients.

### Study Design and Patient Selection

**Figure 1.**
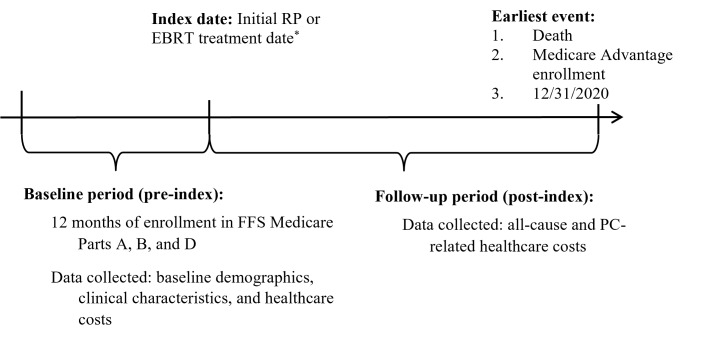
Study Design Abbreviations: EBRT, external beam radiation therapy; FFS, Fee-For-Service; PC, prostate cancer; PC, prostate cancer; RP, radical prostatectomy. *RP or EBRT treatment had to be initiated within 6 months after LPC diagnosis as the patient’s initial definitive therapy.

This was a retrospective, observational, cohort study. The patient selection process is illustrated in **Figure 1**. Patients aged ≥65 years with newly diagnosed LPC between 2012 and 2019 were eligible for inclusion. Patients with LPC were defined by TNM staging system as having a pelvic lymph node status of N0 or N1 and a metastasis status of M0. Eligible patients must have received either EBRT or RP as their initial definitive treatment within 6 months of diagnosis, as determined from Medicare claims. The cohort index date was defined as the earliest observed claim for definitive treatment. To ensure complete capture of treatment history, patients were also required to have continuous enrollment in Medicare Fee-For-Service Parts A, B, and D for ≥12 months prior to the index date (the baseline period). Patients were excluded if they had a history of other primary cancers, evidence of metastatic disease at diagnosis, indeterminate LPC risk classification, or received definitive prostate cancer treatment other than androgen deprivation therapy (ADT) before the index date.

Patients were stratified into high-risk or low/intermediate-risk cohorts according to National Comprehensive Cancer Network criteria, based on T stage, Gleason score, and PSA level at diagnosis.^6^ All patients were followed for economic outcomes from the index date through the earliest event of death, disenrollment from Medicare Fee-For-Service Part A, B, or D, or the end of the study period (31 December 2020).

### Patient Characteristics and Economic Outcomes

Patients’ sociodemographic and prostate cancer–related clinical characteristics were extracted from SEER CANCER files. These included age at the index date, sex, marital status, urban or rural residence, geographic region, and household income and education at the census tract level, as well as TNM stage, PSA results, Gleason score, and histology. Comorbidity burden was assessed during the 12-month baseline period prior to the cohort index date. The National Cancer Institute (NCI) Comorbidity Index was used, which captures 16 noncancer comorbid conditions based on diagnostic codes.^19^ The use of ADT during the baseline period was also extracted from Medicare claims using Current Procedural Terminology codes and Healthcare Common Procedure Coding System codes.

The primary outcomes of interest were post-index all-cause costs and prostate cancer–related costs from the Medicare payer perspective. The total costs were categorized according to Medicare claim type and included emergency department (ED), inpatient, outpatient, other (home health agency, hospice, and durable medical equipment), and pharmacy (Part D) costs. Prostate cancer–related drug costs were also analyzed separately, which included ADT, anti-androgens, poly ADP-ribose polymerase (PARP) inhibitors, other systemic therapies (chemotherapy, immunotherapy, and radium 223 dichloride), and bone metastasis treatments. Second-generation anti-androgens, PARP, and other systemic therapies were analyzed as treatments for advanced prostate cancer.

### Statistical Analysis

All baseline characteristics (at first therapy initiation) were summarized using descriptive statistics. Categorical variables were reported as counts and proportions (%), and continuous variables were reported as means and standard deviations (SD). Cost outcomes were presented on a per-patient-per-year (PPPY) basis. All costs were adjusted to 2023 US dollars using the medical care component of the Consumer Price Index. Two-sample *t*-test was used to compare the mean healthcare costs between cohorts.

## RESULTS

### Baseline Patient Demographic and Clinical Characteristics

**Figure 2.**
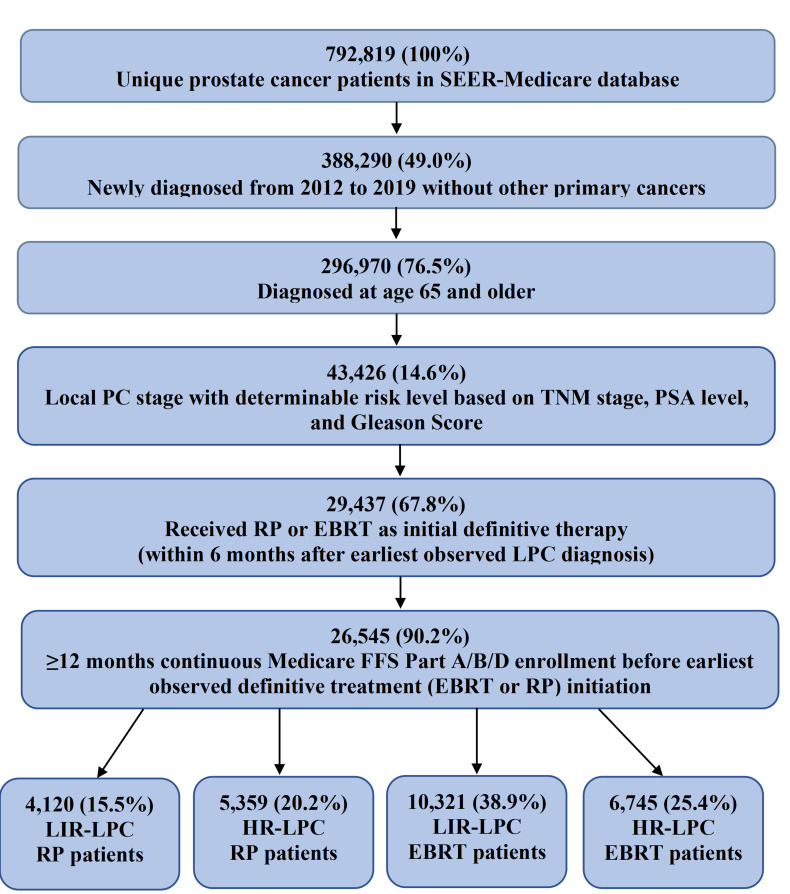
Patient Selection Criteria Abbreviations: EBRT, external beam radiation therapy; FFS, Fee-For-Service; HR-LPC, high-risk localized or locally advanced prostate cancer; LIR-LPC, low or intermediate-risk localized or locally advanced prostate cancer; PSA, prostate-specific antigen; RP, radical prostatectomy; SEER, Surveillance, Epidemiology, and End Results.

A total of 26 545 patients met the selection criteria and were stratified into 4 independent cohorts by risk classification of HR vs LIR and initial treatment of RP vs EBRT (**Figure 2**). Stratification by risk status was performed because disease risk influences treatment approaches and associated healthcare costs. Patient baseline demographic and clinical characteristics are summarized in **Table 1**.

**Table 1.** Demographic and Clinical Characteristics of Patients with LIR-LPC and HR-LPC

	**EBRT Cohorts**		**RP Cohorts**	
**LIR-⁠LPC (N = 10 321)**	**HR-⁠LPC (N = 6745)**	**LIR-⁠LPC (N = 4120)**	**HR-⁠LPC (N = 5359)**	
Age at index date, mean ± SD, years	72.4 ± 4.5	74.4 ± 5.4	69.6 ± 3.3	70.4 ± 3.7
Age category, years, n (%)				
65-69	3198 (31.0)	1375 (20.4)	2305 (56.0)	2550 (47.6)
70-74	3983 (39.0)	2169 (32.2)	1460 (35.4)	2117 (39.5)
75-79	2382 (23.1)	1987 (29.5)	314 (7.6)	593 (11.1)
80-84	677 (6.6)	959 (14.2)	*	77 (1.4)
85+	81 (0.8)	255 (3.8)	*	22 (0.4)
Race, n (%)				
White	8736 (84.6)	5632 (83.5)	3633 (88.2)	4700 (87.7)
Black	961 (9.3)	576 (8.5)	243 (5.9)	282 (5.3)
American Indian/Alaskan Native	24 (0.2)	24 (0.4)	*	13 (0.2)
Asian/Pacific Islander	391 (3.8)	379 (5.6)	200 (4.9)	320 (6.0)
Unknown	209 (2.0)	134 (2.0)	*	44 (0.8)
Ethnicity, n (%)				
Non-Hispanic	9710 (94.1)	6318 (93.7)	3853 (93.5)	4995 (93.2)
Hispanic	611 (5.9)	427 (6.3)	267 (6.5)	364 (6.8)
% with college education, mean ± SD^a^	33.6 ± 19.7	33.7 ± 19.7	34.8 ± 19.5	35.7 ± 20.1
Marital status, n (%)				
Never married	789 (7.6)	535 (7.9)	276 (6.7)	476 (8.9)
Married	7190 (69.7)	4620 (68.5)	3273 (79.4)	4104 (76.6)
Others^b^	2342 (22.7)	1590 (23.6)	571 (13.9)	779 (14.5)
Urban/rural residence^c^, n (%)				
Urban	8668 (84.0)	5567 (82.5)	3512 (85.2)	4572 (85.3)
Rural	1653 (16.0)	1178 (17.5)	608 (14.8)	787 (14.7)
Year of prostate cancer diagnosis, n (%)				
2012	997 (9.7)	482 (7.2)	423 (10.3)	374 (7.0)
2013	1059 (10.3)	561 (8.3)	429 (10.4)	448 (8.4)
2014	1179 (11.4)	700 (10.4)	443 (10.8)	513 (9.6)
2015	1230 (11.9)	838 (12.4)	514 (12.5)	656 (12.2)
2016	1392 (13.5)	922 (13.7)	559 (13.6)	772 (14.4)
2017	1459 (14.1)	1053 (15.6)	620 (15.1)	837 (15.6)
2018	1480 (14.3)	1114 (16.5)	569 (13.8)	865 (16.1)
2019	1525 (14.8)	1075 (15.9)	563 (13.7)	894 (16.7)
Histology, n (%)				
Adenomas and adenocarcinomas	10,285 (99.7)	6687 (99.1)	4092 (99.3)	5288 (98.7)
Others^d^	36 (0.3)	58 (0.9)	28 (0.7)	71 (1.3)
Tumor stage^e^, n (%)				
T0/T1/unknown	7224 (70.0)	3164 (46.9)	161 (3.9)	91 (1.7)
T2	3097 (30.0)	2524 (37.4)	3959 (96.1)	1408 (26.3)
T3	0 (0)	949 (14.1)	0 (0)	3788 (70.7)
T4	0 (0)	108 (1.6)	0 (0)	72 (1.3)
N stage,^5^ n (%)				
N0	10,321 (100)	6426 (95.3)	4120 (100)	4814 (89.8)
N1	0 (0)	319 (4.7)	0 (0)	545 (10.2)
PSA level at diagnosis, n (%)				
<10 ng/mL	8149 (79.0)	2939 (43.6)	3543 (86.0)	3369 (62.9)
10-20 ng/mL	2172 (21.0)	1603 (23.8)	577 (14.0)	1074 (20.0)
>20 ng/mL	0 (0)	1873 (27.8)	0 (0)	595 (11.1)
Missing	0 (0)	330 (4.9)	0 (0)	321 (6.0)
Gleason score, n (%)				
2-6	2609 (25.3)	139 (2.1)	596 (14.5)	56 (1.0)
7	7712 (74.7)	937 (13.9)	3524 (85.5)	2023 (37.8)
8-10	0 (0)	5626 (83.4)	0 (0)	3266 (60.9)
Missing	0 (0)	43 (0.6)	0 (0)	14 (0.3)
NCI comorbidity score^f^, mean ± SD	1.5 ± 1.7	1.8 ± 1.9	1.1 ± 1.4	1.3 ± 1.5
Common individual comorbidities				
Hypertension	8204 (79.5)	5444 (80.7)	2930 (71.1)	3907 (72.9)
Diabetes	3313 (32.1)	2408 (35.7)	1019 (24.7)	1442 (26.9)
Chronic pulmonary disease	1946 (18.9)	1379 (20.4)	627 (15.2)	904 (16.8)
Peripheral vascular disease	1821 (17.6)	1503 (22.3)	518 (12.6)	823 (15.4)
Obesity	1699 (16.5)	1246 (18.5)	580 (14.1)	850 (15.9)
Cerebrovascular disease	1407 (13.6)	997 (14.8)	392 (9.5)	535 (10.0)
Renal disease	1346 (13.0)	1088 (16.1)	359 (8.7)	522 (9.7)
ADT use prior to index date	2922 (28.3)	4828 (71.6)	71 (1.7)	276 (5.2)
No concurrent use on index date	6712 (65.0)	973 (14.4)	4046 (98.2)	4724 (88.2)
Concurrent use on index date	3609 (35.0)	5772 (85.6)	74 (1.8)	635 (11.9)
Duration of follow-up period, mean ± SD, months	45.6 ± 26.6	41.0 ± 24.5	47.7 ± 27.3	43.8 ± 25.8

Abbreviations: EBRT, external beam radiation therapy; HR-LPC, high-risk localized or locally advanced prostate cancer; LIR-LPC, low or intermediate-risk localized or locally advanced prostate cancer; NCI, National Cancer Institute; RP, radical prostactectomy; SD, standard deviation.

Asterisks denote values of ≤10, suppressed according to the Centers for Medicare & Medicaid Services cell size suppression policy.

^a^Education at census tract level.

^b^Others include separate, divorces, widowed, unmarried or domestic partner, and unknown.

^c^Urban includes counties in metropolitan areas with populations of 1 million or more, 250 000 to 1 million, or fewer than 250 000. Rural includes areas with a population of 20 000 or more either adjacent to or not adjacent to a metro area; populations of 2500 to 19 999 either adjacent to or not adjacent to a metro area; and completely rural areas or those with fewer than 2500 people, also either adjacent to or not adjacent to a metro area.

^d^Others include unspecified neoplasms, epithelial neoplasms, NOS, squamous cell neoplasms, basal cell neoplasms, cystic, mucinous and serous neoplasms, ductal and lobular neoplasms, acinar cell neoplasms, and complex epithelial neoplasms.

^e^TNM is a classification system used for many cancer types to describe the amount and spread of cancer. T describes tumor size and any spread to nearby tissue; N describes cancer spread to nearby lymph nodes; M describes cancer metastasis. The numbers denotes the spread and metastasis of the cancer, with Stage 0 reflects minimal involvement, usually carcinoma in-situ, whereas Stage 4 indicates either greatest tumor involvement or distant metastasis.^4^

^f^ https://healthcaredelivery.cancer.gov/seermedicare/considerations/comorbidity.html

Among the 17 066 patients treated with EBRT, 60% (N = 10 321) were low or intermediate risk and 40% (N = 6745) were high risk. The mean age on index date was approximately 73 years. The majority (>83%) of patients were White. The mean NCI comorbidity score was 1.5 and 1.8, respectively, for the LIR-LPC and HR-LPC cohorts. Among the 9479 patients treated with RP, 43% (N = 4120) had LIR-LPC and 57% (N = 5359) had HR-LPC. The mean age on index date was approximately 70 years. Most (>87%) of the patients were White. Compared with the corresponding EBRT cohorts, patients in the RP cohorts appeared to have less comorbidity burden, with mean NCI comorbidity scores of 1.1 and 1.3, respectively, for the LIR-LPC and HR-LPC patients. Across cohorts, the most common comorbidities were hypertension and diabetes, followed by chronic pulmonary disease, peripheral vascular disease, and obesity. Among patients treated with EBRT, 28.3% of LIR-LPC patients and 71.6% of HR-LPC patients received ADT prior to the index date, while among patients treated with RP, 1.7% of LIR-LPC and 5.2% of HR-LPC patients received ADT.

After EBRT initiation, LIR-LPC patients had a mean follow-up time of 46 months while HR-LPC patients had a mean follow-up time of 41 months. After RP initiation, the mean follow-up time was 48 months for LIR-LPC patients and 44 months for HR-LPC patients, respectively.

### All-Cause Healthcare Costs

All-cause healthcare costs across cohorts during the follow-up period are summarized in **Table 2**.

Among patients treated with EBRT, the mean all-cause healthcare cost in the HR-LPC cohort was $9837 higher than that in the LIR-LPC cohort ($34 441 vs $24 604 PPPY; *P* < .0001). Similarly, among patients treated with RP, the mean all-cause PPPY healthcare cost was $6829 higher in the HR-LPC cohort than in the LIR-LPC cohort (mean, $23 820 vs $16 991 PPPY; *P* < .0001). Across the EBRT and RP cohorts, the largest proportion of all-cause healthcare costs was attributed to outpatient cost, followed by inpatient and prescription drug costs. Other components, including ED visits, other medical care, and prescription drug costs, were also significantly higher in HR-LPC patients.

**Table 2.** All-Cause Healthcare Costs of Patients with LIR-LPC vs HR-LPC During Follow-up Period

	**EBRT Cohorts, PPPY, Mean ± SD^a^**			**RP Cohorts, PPPY, Mean ± SD^a^**		
**LIR-LPC (N = 10 321)**	**HR-LPC (N = 6745)**	***P***	**LIR-LPC (N = 4120)**	**HR-LPC (N = 5359)**	***P***	
Person-year	39 724	23 345		16 562	19 793	
All-cause healthcare costs	24 604 ± 27 646	34 441 ± 37 337	<.0001	16 991 ± 27 865	23 820 ± 26 437	<.0001
Emergency department	239 ± 569	339 ± 715	<.0001	185 ± 488	207 ± 432	.027
Inpatient	4881 ± 16 338	7463 ± 23 266	<.0001	6922 ± 22 526	7308 ± 14 828	.3412
Outpatient	16 505 ± 14 017	21 408 ± 16 739	<.0001	7994 ± 8807	12 759 ± 12 870	<.0001
Other medical care^b^	756 ± 2665	1331 ± 3797	<.0001	492 ± 1864	669 ± 2460	<.0001
Prescription drugs	2223 ± 7779	3900 ± 11 167	<.0001	1398 ± 4670	2878 ± 9592	<.0001

Abbreviations: EBRT, external beam radiation therapy; HR-LPC, high-risk localized or locally advanced prostate cancer; LIR-LPC, low or intermediate-risk localized or locally advanced prostate cancer; PPPY, per-person-per-year; RP, radical prostatectomy; SD, standard deviation.

^a^Cost data are adjusted to 2023 US dollars.

^b^Includes home health agency, hospice, durable medical equipment.

### Prostate Cancer-Related Healthcare Costs

Prostate cancer–related healthcare costs and prostate cancer treatment costs for LIR-LPC and HR-LPC patients in the EBRT and RP cohorts during the follow-up period are summarized in **Table 3**.

**Table 3.** Prostate Cancer–Related Healthcare Costs of Patients with LIR-LPC vs HR-LPC During Follow-up Period

	**EBRT Cohorts PPPY Mean ± SD^a^**			**RP Cohorts PPPY Mean ± SD^a^**		
**LIR-LPC (N = 10 321)**	**HR-LPC (N = 6745)**	***P***	**LIR-LPC (N = 4120)**	**HR-LPC (N = 5359)**	***P***	
Person-year	39 724	23 345		16 562	19 793	
Prostate cancer–related healthcare costs	12 385 ± 14 663	20 211 ± 25 905	<.0001	7 264 ± 19 933	13 354 ± 17 935	<.0001
Emergency department	21 ± 146	49 ± 225	<.0001	10 ± 114	23 ± 135	<.0001
Inpatient	833 ± 7018	2 440 ± 15 175	<.0001	3902 ± 18 907	4130 ± 9485	0.4782
Outpatient	11 258 ± 11 598	15 564 ± 14 310	<.0001	3206 ± 4653	7746 ± 9678	<.0001
Other medical care^b^	117 ± 930	455 ± 2298	<.0001	99 ± 714	229 ± 1524	<.0001
Prescription drugs	156 ± 2189	1704 ± 9136	<.0001	47 ± 1378	1226 ± 7652	<.0001
Prostate cancer–related drug costs	429 ± 3129	3415 ± 12 128	<.0001	100 ± 1849	2053 ± 10 200	<.0001
Androgen deprivation therapy	189 ± 547	1072 ± 1108	<.0001	23 ± 148	337 ± 741	<.0001
Anti-androgens (1st and 2nd generations)	140 ± 2146	1602 ± 8766	<.0001	46 ± 1378	1187 ± 7517	<.0001
PARP inhibitor	0 ± 0	42 ± 1357	.0112	0 ± 0	12 ± 539	.0915
Other systemic therapy^c^	58 ± 1227	478 ± 4734	<.0001	21 ± 613	377 ± 3625	<.0001
Bone treatment	42 ± 498	221 ± 1182	<.0001	10 ± 191	139 ± 1043	<.0001
Advanced treatment^d^	197 ± 2769	2114 ± 11 274	<.0001	67 ± 1672	1574 ± 9361	<.0001
RP-related cost^e^	56 ± 603	87 ± 865	.01	4983 ± 18 848	5083 ± 8384	.7513
EBRT-related cost^e^	8691 ± 10 235	10 968 ± 11 094	<.0001	461 ± 2040	2840 ± 5209	<.0001

Abbreviations: EBRT, external beam radiation therapy; HR-LPC, high-risk localized or locally advanced prostate cancer; LIR-LPC, low- or intermediate-risk localized or locally advanced prostate cancer; PARP, poly(ADP-ribose) polymerase; PPPY, per-person-per-year; RP, radical prostatectomy; SD, standard deviation.

^a^Cost data are adjusted to 2023 US dollar.

^b^Includes home health agency, hospice, durable medical equipment.

^c^Includes chemotherapy, immunotherapy, and radium 223 dichloride.

^d^Includes second generation anti-androgens, PARP inhibitor, and other systemic therapy.

^e^Includes RP- and EBRT-related costs used as adjuvant or salvage treatment.

Among patients treated with EBRT, the mean prostate cancer–related healthcare cost in the HR-LPC cohort was $7826 higher than in the LIR-LPC cohort ($20 211 vs $12 385 PPPY; *P* < .0001), with the largest proportion of the cost attributable to outpatient cost. Prostate cancer–related outpatient cost was $4306 higher in the HR-LPC cohort compared with the LIR-LPC cohort (mean, $15 564 vs $11 258 PPPY; *P* < .0001). Among patients treated with RP, the mean prostate cancer–related cost was $6090 higher in the HR-LPC cohort than in the LIR-LPC cohort ($13 354 vs $7264 PPPY, *P* < .0001). While the mean inpatient ($3902) and outpatient ($3206) costs were comparable in the LIR-LPC cohort, outpatient cost accounted for the largest proportion of total prostate cancer–related healthcare cost in the HR-LPC cohort ($7746) and was significantly higher than that in the LIR-LPC cohort. Across the EBRT and RP cohorts, HR-LPC patients also incurred significantly higher ED, other medical care, and prescription drug costs.

Among patients treated with EBRT, the mean prostate cancer–related drug cost was $2986 higher in the HR-LPC cohort compared with the LIR-LPC cohort ($3415 vs $429; *P* < .0001). Among patients treated with RP, the mean drug cost was also higher in the HR-LPC cohort, with a difference of $1953 ($2053 vs $100; *P* < .0001). For both treatment types, HR-LPC patients incurred significantly higher ADT, anti-androgens, other systemic therapy, bone treatment costs. The advanced PC treatment costs were also significantly higher in HR-LPC patients for both treatment types ($2114 vs $197 in EBRT; $1574 vs $67 in RP; *P* < .0001).

## DISCUSSION

In this real-world study, we analyzed all-cause and prostate cancer–related healthcare costs for Medicare beneficiaries diagnosed with LPC, treated with EBRT or RP from 2012 to 2019. The analyses found that HR-LPC patients incurred significantly higher all-cause and prostate cancer–related healthcare costs than LIR-LPC patients, regardless of whether they received EBRT or RP as the initial definitive therapy. This observation is consistent with findings from limited prior research on the economic impact of LPC, which indicate that high-risk disease is associated with significantly increased healthcare costs.^8^ Gustavsen et al estimated average 10-year direct medical costs associated with the management of LPC to be $45 957, $99 445, and $188 928 for low, intermediate, and high-risk patients, respectively.^8^ The study also reported that HR-LPC patients incurred significantly higher cumulative cost per patient.^8^

The increased healthcare costs for HR-LPC patients can be attributed to several factors. First, HR-LPC patients in this analysis were older at the index date and had more comorbidities, potentially leading to higher healthcare utilization and costs. Second, treating HR-LPC is more complex than that of LIR-LPC and often requires more intensive and multimodality treatment, longer therapy durations, and more frequent monitoring.^20–22^ This also adds to the significant healthcare costs.^23^ In addition, our previous study investigating clinical outcomes in LPC patients receiving EBRT as initial therapy found that HR-LPC patients had significantly worse clinical outcomes than LIR-LPC patients, including lower 5-year metastasis-free survival (70.5% vs 87.3%; *P* < .001) and overall survival (66.5% vs 83.3%; *P* < .001), more frequent initiation of advanced prostate cancer treatment (49.7% vs 21.1%), and more common concurrent ADT use (97.0% vs 84.2%) for a longer mean duration (22.0 vs 12.2 months).^24^ These differences in clinical outcomes and treatment patterns likely contributed to the elevated costs observed in the current analysis.

As advanced-stage prostate cancer diagnoses continue to rise, findings from this study have important implications for healthcare resource planning and policy decision-making. The substantially higher costs observed among patients with HR-LPC highlight the greater economic burden of HR-LPC and may help health systems and payers anticipate future resource needs. In addition, these real-world cost estimates provide a benchmark for evaluating budget impact and economic value of emerging treatment strategies. Such information may inform reimbursement decisions, healthcare planning, and assessments of whether new interventions can reduce the long-term economic burden of prostate cancer. Future studies should assess the value of emerging therapies and include broader economic impacts, including patient out-of-pocket costs and caregiver burden.

### Strengths and Limitations

This study contributes valuable evidence on the economic burden of LPC stratified by initial definitive treatment and risk classification, a topic with limited prior investigation. One of the strengths of this study is its comprehensive analysis of healthcare cost categories for LPC, which compared both all-cause and PC-related expenses between HR-LPC and LIR-LPC patients and identified cost patterns across different risk cohorts. By leveraging the SEER–Medicare database, this study also ensured a population-based sample of LPC patients, enhancing the generalizability of the findings.^16^

Nonetheless, there are several limitations in this study. First, this study was retrospective and had limitations inherent to administrative data, including potential coding errors that could lead to misclassification of diagnosis and clinical outcomes. Treatments paid by patient themselves or through other insurances may not be captured. Secondly, this study was conducted from the Medicare payer perspective and did not account for patients’ out-of-pocket costs or healthcare costs reimbursed outside of Medicare coverage. Furthermore, indirect costs, such as productivity loss due to absenteeism or caregiver burden were also not included. Third, our study is descriptive in nature and does not break down the impact on costs by individual clinical and social economic attributes. However, patients are rarely diagnosed and treated without accompanying comorbidities and socioeconomic factors. The primary goal of the current study is to quantify the overall cost burden among the target LPC population, reflecting the combined impact of patients’ clinical characteristics, initial treatment options, and broader socioeconomic context, rather than estimating the contribution of risk classification alone. Lastly, because the study focused on Medicare FFS population, the findings may not be generalizable to Medicare advantage or commercially insured patient populations.

## CONCLUSION

Our study quantified the substantial economic burden in Medicare beneficiaries diagnosed with LPC, particularly among HR-LPC patients. HR-LPC patients were associated with significantly higher all-cause and prostate cancer–related healthcare costs than those with LIR-LPC, regardless of whether they received EBRT or RP. These elevated costs are likely to reflect the greater treatment intensity, longer therapy duration, and increased follow-up required for HR-LPC. The substantial cost differences between LIR-LPC and HR-LPC cohorts emphasize the unmet need for novel treatment strategies that can reduce disease progression and ultimately reduce the healthcare costs of LPC and the economic burden of prostate cancer care. Future research is needed to evaluate whether emerging treatment strategies that improve clinical outcomes can reduce healthcare utilization and costs among patients with HR-LPC.

### Disclosures

L.K. is an employee of AdventHealth Urology Denver and has received consulting fees from Johnson & Johnson. S.D. and J.H. are employees of Johnson & Johnson and stockholders of Johnson & Johnson. N.S. is an employee of Carolina Urologic Research Center and has received consulting fees from Johnson & Johnson. K.J. was an employee of Johnson & Johnson at the time the manuscript was written.

## Data Availability

The datasets used to conduct this study are available upon approval of a research protocol from the National Cancer Institute. Instructions for obtaining these data are available at https://healthcaredelivery.cancer.gov/seermedicare/obtain/.
